# Signing protein–protein interaction networks

**DOI:** 10.1093/bioinformatics/btaf674

**Published:** 2025-12-22

**Authors:** Lorenzo Federico Signorini, Martin Kupiec, Roded Sharan

**Affiliations:** Blavatnik School of Computer Science and AI, Tel Aviv University, Tel Aviv 6997801, Israel; Schmunis School of Biomedicine and Cancer Research, Tel Aviv University, Tel Aviv 6997801, Israel; Schmunis School of Biomedicine and Cancer Research, Tel Aviv University, Tel Aviv 6997801, Israel; Blavatnik School of Computer Science and AI, Tel Aviv University, Tel Aviv 6997801, Israel

## Abstract

**Motivation:**

Protein–protein interactions (PPIs) provide the skeleton for signaling pathways in the cell. Their experimental measurement, however, reveals only the existence of an interaction without any information on its functional roles. A key step in developing a working logical model of cell signaling is annotating activation/repression (sign) of an interaction.

**Results:**

Here, we develop SIGN Annotation aLgorithm (SIGNAL), a method for annotating PPI networks with signs based on cause-effect data. The approach is based on a multiplicative model in which the effect of a pathway is assumed to be the product of the signs along its edges. The algorithm uses network propagation techniques to quantify the influence of each edge on gene expression changes, and the resulting features are fed to a classifier for sign prediction. We validate our method using known annotations and demonstrate the utility of SIGNAL for predicting the effect of a knockout on gene expression and on telomere length.

**Availability and implementation:**

SIGNAL code is available at https://github.com/L-F-S/PPI_Network_Signer.

## 1 Introduction

The problem of annotating protein–protein interactions (PPIs) and subsequent modeling of signaling-regulatory pathways has intrigued bioinformaticians since 2004, when a first such model (called physical network model) was developed by Yeang *et al.* (2004). In their paper, Yeang *et al.* proposed a simple yet effective way to model the effect of a molecular interaction (activation or repression) as a plus or minus sign: physical interactions are directed and signed and, the effect (sign) of a signaling cascade is equal to the product of the signs along its interactions. Another early work (Gat-Viks *et al.* 2006) introduced the combinatorial analog concept of chain functions and proposed a way to reconstruct such functions by using a minimal number of knockout experiments. They demonstrated the application of their method to the reconstruction of the yeast galactose utilization network.

Since then, the availability of data has increased dramatically, and high-throughput technologies such as yeast two-hybrid (Caufield *et al.* 2012, Luck *et al.* 2020), co-immunoprecipitation followed by mass spectrometry (Free *et al.* 2009), and affinity purification mass spectrometry (Huttlin *et al.* 2017) are allowing for systematic screening of hundreds of thousands of physical PPIs, which are deposited into large network databases (Orchard *et al.* 2014, Oughtred *et al.* 2019, Signorini *et al.* 2021). Such interactomes are fundamental tools to achieve a comprehensive depiction of the cellular machinery. Nevertheless, translating these networks into a functional simulation of cellular behavior under diverse genetic and environmental stimuli remains a formidable challenge, calling for the development of adequate computational methods to parse and harvest useful information for complex biological systems modeling. One crucial limit to the predictive power of these networks is the lack of information on the logic of the interactions, namely the *direction* of signal flow and its activation/repression effect (*sign*). At present, only a few well-studied pathways have direction and sign information available (Patkar and Sharan 2018). However, it is expected that a large fraction of the PPIs could admit such an annotation (Silberberg *et al.* 2014). Acquiring this annotation information is essential as a prerequisite for developing any coherent model of a system under investigation.

Here, we focus on the task of *sign* annotation to physical interactions. Peleg and co-workers (Peleg *et al.* 2010) demonstrated the NP-hardness of the sign assignment problem and devised network-free algorithms to predict the genome-wide effects of gene knockouts. The task of inferring physical interaction signs on a network while considering paths of any length was first addressed by Houri and Sharan (Houri and Sharan 2012). They specifically aimed to find an assignment that maximized the number of pairs having a path with the required sign. Additionally, they introduced network reduction techniques and an integer linear programming (ILP) formulation to efficiently solve this problem on existing physical interaction networks. Later, Patkar and Sharan (2018) proposed a novel set of models based on a network-based ILP framework, which circumvents the issue of network reduction of previous works, resulting in a substantial upscaling of predictive capacity.

In this work, we developed the SIGNAL (SIGN Annotation aLgorithm) method for sign annotation in PPI networks based on cause-effect data. Following Yeang *et al.* (2004), we assume a multiplicative sign model in which the effect of a pathway is the product of the signs along its edges. We build on the observation that, in such a model, a single negatively signed edge is enough to revert the effect sign of the path it occurs in while positively signed edges do not affect path signs. It is therefore reasonable to assume that negative (inhibitory) interactions are less frequent than positive (activating) ones in physical interaction networks. By propagating a gene expression signal from a pathway starting point across an interaction network, and comparing the results to a similar propagation obtained after removing individual edges, we can deduce which edges are negative, as they have a greater effect than positive ones. To measure the influence of an edge, we quantify how much flow passes through edges on its way from causal to affected genes using network propagation techniques. The resulting features are then fed to a classifier for sign prediction. We show that SIGNAL outperforms the state of the art (Patkar and Sharan 2018) in sign prediction accuracy. Additionally, we validate our model by reconstructing knockout signatures use SIGNAL, and by predicting the telomere length phenotype of the yeast telomere length maintenance (TLM) mutants. Finally, we showcase the potential usage of SIGNAL in unveiling the potential mechanism of actions of protein import into organelles, through functional enrichment of predicted negative edges and network reconstruction.

## 2 Materials and methods

### 2.1 The signaling model

SIGNAL is based on a simple yet effective signal transduction model (Yeang *et al.* 2004), where molecular interactions of a signaling cascade are represented by directed edges, and their effect, which can be either activation or repression, is represented by a plus or a minus sign. The model further states that the final effect *s_out_* of a pathway of *n* edges and *n + 1* nodes, is equal to the product of the signs *s_i_* along its edges:


(1) sout=∏insi

Assuming this multiplicative interaction model, a single negative edge suffices to revert its overall effect. Therefore, we hypothesize that negative interactions are less prevalent than positive ones in physical interaction networks. Furthermore, the removal of a negative edge is expected to have a greater effect on the system’s overall state than the removal of a positive edge. This asymmetry between activation and inhibition provides a measurable signal that can be exploited to train a classifier capable of distinguishing between the two interaction types.

### 2.2 Input data

Our model builds on three main types of experimental data:

1. *Base network.* Although the original physical network model assumes directed signaling flow, our framework generalizes sign inference to an undirected PPI network. of 151 717 (yeast) and 545 673 (human) undirected experimentally measured PPIs, taken from (Signorini *et al.* 2021). All gene IDs were converted to Entrez IDs. If not already present, proteins from the signed and knockout signatures datasets were integrated into the base network, together with their information on interaction directionality. The final base networks consist of 316 142 (yeast) and 1 091 346 (human) directed edges, where each undirected edge is represented as two oppositely directed edges. The quick brown fox jumps over the lazy dog. The quick brown fox jumps over the lazy dog.
2. *Knockout signatures.* These are cause-effect data, represented by sets of source genes (knockouts) and their functional targets. Each of the target genes carries a sign representing the direction of its expression change after inhibition (via knockout or other experimental perturbation) of source gene. Yeast knockout signatures are extracted from a set of transcriptional signatures from knockout mutants from (Kemmeren *et al.* 2014)., resulting in 235 knockouts and 1000 targets (Section 2, available as supplementary data at *Bioinformatics* online). For humans, signatures come from genome-wide perturb-seq data on K562 myelogenous leukemia cell lines from (Replogle *et al.* 2022). Only strong perturbations as defined in the work were retained for a total of 1973 perturbations *(sources)* and 2321 genes (*targets*) (Section 2, available as supplementary data at *Bioinformatics* online).
3. *Signed interactions.* These are PPIs for which the sign of the interaction effect (activating/repressing) is known. They represent the training data of our model and were taken from the following sources (Table 1):
  - KEGG signaling pathways (Kanehisa and Goto 2000, Patkar and Sharan 2018). The following labels were regarded as positive signs in PPIs: “association/binding,” “activation,” “expression.” And as negative: “inhibition,” “dissociation” for a total of 189 interactions (yeast) and 1303 interactions (human).
  - Kinase/Phosphatase interaction datasets (KPI). Phosphorylation (kinase/substrate) interactions were taken as positive, and dephosphorylation (phosphatase/substrate) as negative. For yeast, the KPI dataset from (Patkar and Sharan 2018) was used, for a total of 1072 interactions, while for humans 2506 experimentally determined kinase-substrate interactions were taken from PhosphositePlus (Hornbeck *et al.* 2012) and 923 manually curated active phosphatase-substrate interactions were collected from DEpod dataset (Damle and Köhn 2019).
  - UbiNet 2.0 (Li *et al.* 2021): a ubiquitination dataset of E3/substrate interactions. E3 is a ligase in the ubiquitination cascade, which binds the substrate and catalyzes the transfer of ubiquitin to the substrate. Since ubiquitination leads in many cases to the eventual destruction of the target protein, these interactions were annotated as negative, for a total of 211 interactions for yeast and 1272 interactions for human.

**Table:** **Table 1.** Signed datasets size and sign distribution.

Species	Signed interaction dataset	# Positive interactions	# Negative interactions
*S. cerevisiae*	KEGG	136	53
*S. cerevisiae*	KPI	893	179
*S. cerevisiae*	UbiNet2	–	211
*H. sapiens*	KEGG	1096	208
*H. sapiens*	KPI	2506	923
*H. sapiens*	UbiNet2		1272

### 2.3 Features construction

**Figure 1.**
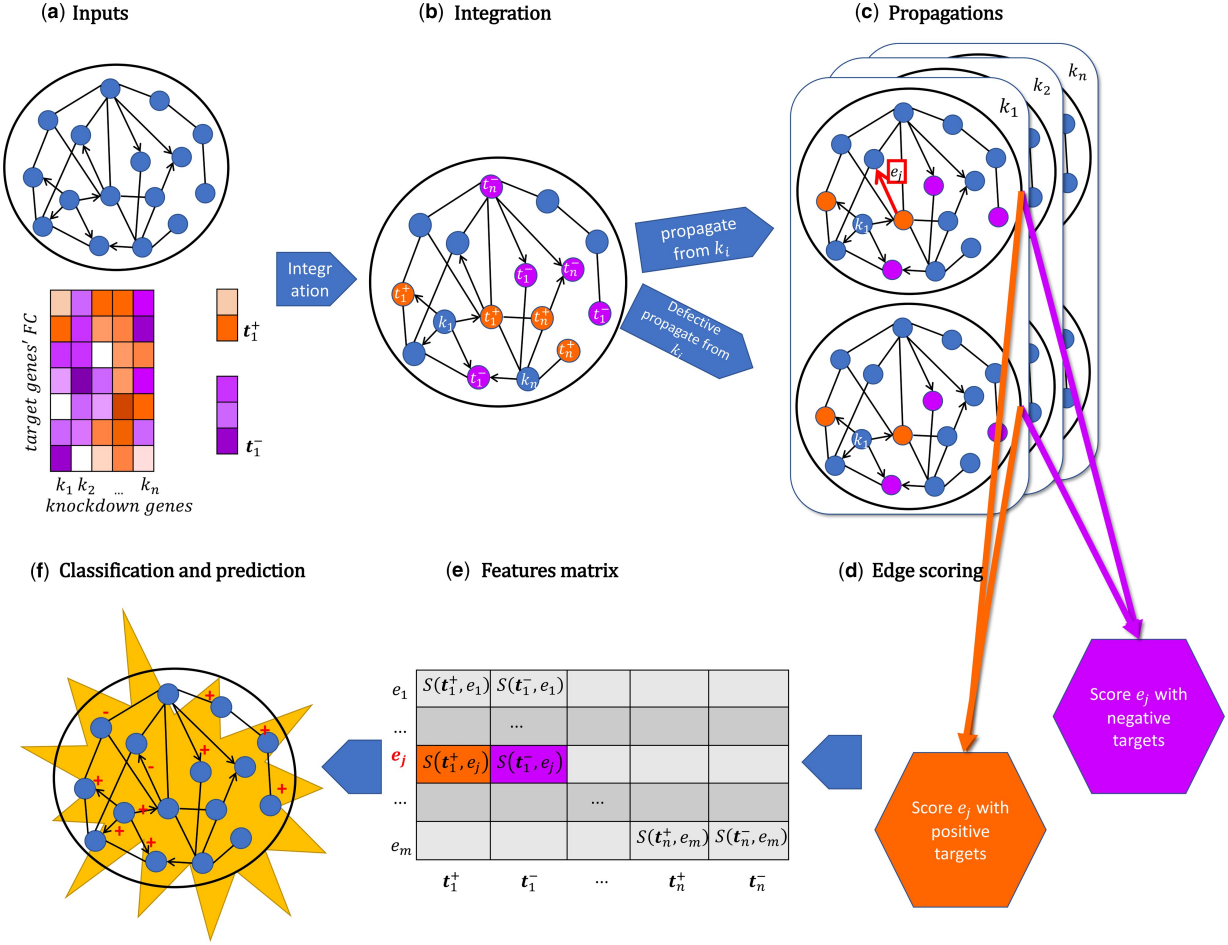
Pipeline of SIGNAL. (a) Inputs for the algorithm are a deletion signature matrix, where for every knockout gene, *k*, a set of differentially expressed genes and their sign of expression change are reported, and a PPI network. (b) Knockout genes *k* and their targets *t* are integrated in the PPI network. Only information about the effect (positive or negative) on the target gene is retained. (c) Information from every causal gene *k* is propagated across all the network twice, first on the original network, and then on a defective network where an edge of interest was removed. (d) Original and defective propagations scores for positive and negative deletion targets are combined separately into two scores for every edge. (e) These scores are used to populate the features matrix. (f) A classifier is trained on edges with known signs to predict signs of unannotated edges in the network.

Our strategy to harness the expected lower frequency of negative interactions is the following: we first quantify the amount of information flow on the network from knockout genes to their targets using propagation (Section 1, available as supplementary data at *Bioinformatics* online). Subsequently, we remove one annotated edge at a time from the full network, thus creating a *defective network* (for a specific edge), and re-measure the same information flow on the defective network. We then build a score *F* that quantifies the difference in information flow between the full network and the defective network. We expect these scores to behave differently if the defective network was built by the removal of a positive or a negative edge. Therefore, we can use these scores as features for a binary sign classifier. Specifically, our model outputs the probability of a given edge having a negative sign. An overview of SIGNAL is shown in Fig. 1.

Features are constructed as follows: each knocked-out gene *k* from the knockout signatures data is associated with a set $t_{k}$ of target genes, which can be divided into two sets, $t_{k}^{+}$ and $t_{k}^{-}$, of genes with positive and negative fold change measured following the knockout of *k*, respectively. Information from node *k* is then propagated (Cowen *et al.* 2017) across the base network, to obtain a vector $P_{k}$ of propagation scores for each node in the network.

For the genes set $t_{k}^{+}$, and for each PPI interaction of interest, *e*, we can calculate a score *F* such that:


(2) F(tk+,e)=A∑t∈t+ΔPk(t,e)|tk+|

where ΔPk(t)=[Pk(t)-Pdk(t,e)] is the difference in propagation scores from knockout *k* for node *t* between normal $P_{k}(t)$ and defective $Pd_{k}(t,e)$ propagation, and $\left|t_{k}^{+}\right|$ is the number of positively affected proteins for knockout *k*. The *defective propagation* Pd(t,e) is calculated by setting the weights of the edge to 0 in both directions in the original adjacency matrix before matrix normalization.

The same formula is applied to propagation scores from negatively affected genes, resulting in two features per knockout experiment, for a total of *2n* features.


(3) F(e)={F(t1+,e), F(t1-,e),…,F(tk+,e), F(tk-,e),…,F(tn+,e),F(tn-,e)}

### 2.4 Classification and evaluation

The feature construction process is repeated for every edge in the training data and the resulting features are used to train a random forest classifier with default parameters from *scikit-learn* (Pedregosa *et al.* 2011). The random forest predicts the probability of each edge to have a negative sign. This final probability is the SIGNAL score, **S.** S-score accuracy was tested with a 5-fold cross-validation. Results of cross-validation are evaluated using the areas under the precision-recall and ROC curves.

Since kinases and phosphatases (hereafter collectively referred to as “enzymes”) often have multiple substrate targets, naïve cross-validation could lead to data leakage if the same enzyme has edges connecting it to substrates in different cross validation folds. To prevent such overfitting effects, the folds were stratified by enzyme, so that edges associated with a single enzyme were all in the same fold. Enzyme groups were then balanced so that the sum of the enzyme degrees was comparable across folds, and each fold contained both kinases and phosphatases.

### 2.5 SIGNAL prediction and phenotype reconstruction

To validate the predictive power of SIGNAL, we built a network model from S scores with the aim of recapitulating experimental phenotypes or signatures. The model reconstructs the sign of a pathway within the network, with an expected binary outcome (i.e. up or down regulation of downstream genes). The reconstruction starts from the signed network, where each edge has a probability S of being negative. For each experimentally annotated cause–effect gene pair, all shortest paths (SPs) connecting the causal gene (*k*) to the affected gene (*t*) are extracted. Each edge *e* within the SP is assigned a sign $s_{e}$ based on a threshold τ and an offset error $\varepsilon_{\tau }$, on the SIGNAL score $S_{e}$ [Equation (4)]:


(4) se={-1 if  Se>τ+ε+1  if  Se<τ+ε

$\varepsilon_{\tau }$ controls confidence in sign assignment. Parameters were set to τ=0.5 and $\varepsilon_{\tau }=0.01$ for all analyses. Edges with $S_{e}\in (\tau -\varepsilon_{\tau },\;\tau +\varepsilon_{\tau })$ are considered unsigned and excluded.

For each SP, the overall sign equals the product of its edge signs, following Equation (1). Consequently, paths with an odd number of negative edges are classified negative, while those with an even number as positive. The contribution of all SPs between a (*k, t)* pair is aggregated into a negativity score:


(5) NegScore(k,t)=|neg(SP(τ))||SP(τ)|

representing the fraction of negative paths among all shortest paths. To reduce noise, only pairs connected by at least 100 paths were considered.The resulting *NegScores* form a probability vector directly comparable to experimental annotations of pathway outcomes. We used variations of this method to reconstruct (*k,t*) pairs and to predict TLM phenotype (see Results).

### 2.6 Functional enrichment analysis

We performed functional enrichment at the interaction level. First, we split the edges in positive and negative edges, following Equation (4), and collected genes adjacent to the interactions Secondly, we selected all GO terms with 10 to 100 genes, to focus on biologically interpretable functional categories. We then assessed gene set enrichment o with hypergeometric test, using the *scipy.stats.hypergeom.sf* function (Virtanen *et al.* 2020) in python 3.9. *P* values were adjusted for multiple hypothesis testing using Bonferroni method as implemented in python’s *statsmodels.stats.multitest* library.

## 3 Results

SIGNAL is an algorithm to annotate the signs of physical interactions in PPI networks, by propagating signals from knockout signatures across the full network and a defective network (see Section 2.3), and integrating the two into a single score. Every individual knockout signature produces one score which is then used as a feature for a binary classifier that predicts the probability of a given interaction to be negative. The algorithm was implemented in Python3.9, and an overview of SIGNAL is shown in Fig. 1. A network annotated with SIGNAL can be seen in Fig. 5.

**Figure 2.**
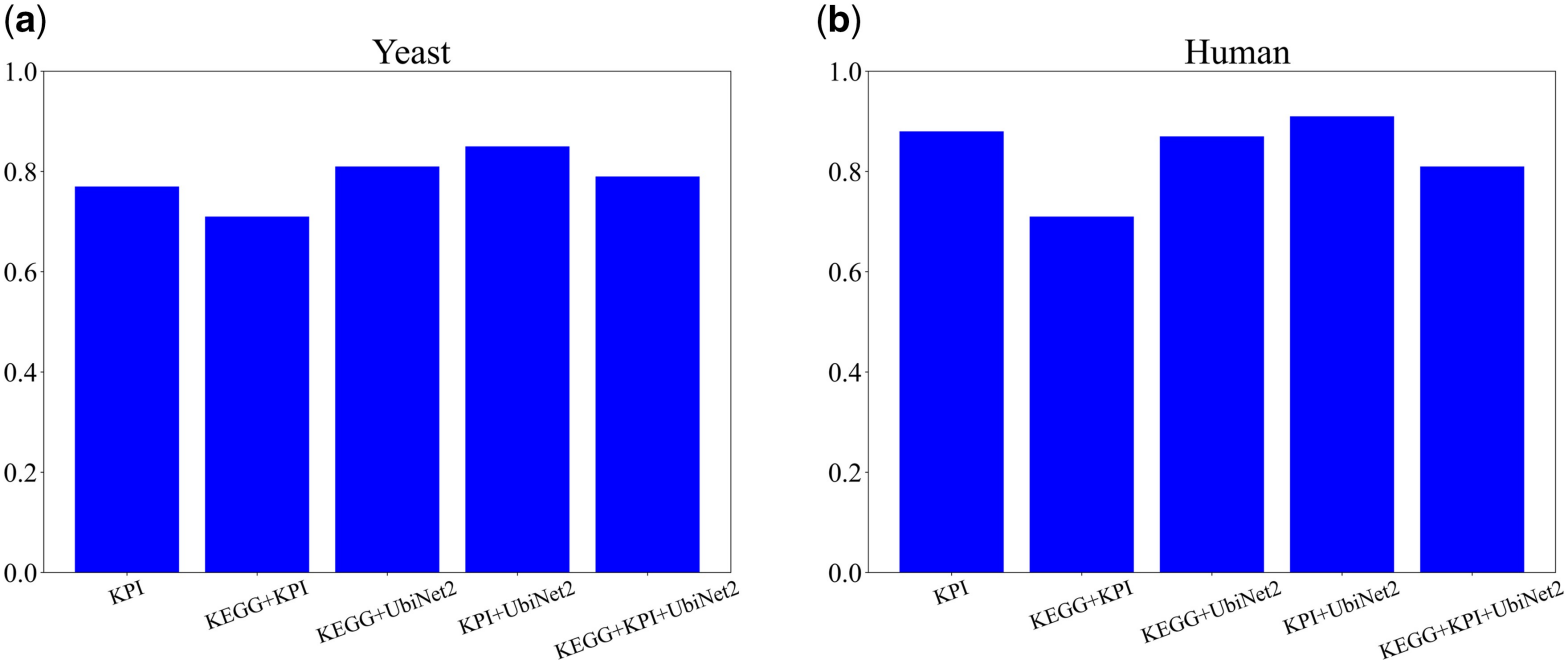
AUC values for 5-fold cross-validations with different datasets for yeast (a) and human (b) datasets.

The signed data are consistent with our initial assumption that negative interactions are less common than positive, since in both human and yeast we see a prevalence of positive interactions (Table 1). Signed edges were used as training data, and features were built for each one of these edges. A random forest classifier was applied to these features and its accuracy was calculated in a 5-fold cross-validation setting. The algorithm’s performance across multiple annotation sources is depicted in Fig. 2.

### 3.1 Comparison with previous state of the art

State of the art results for sign prediction tasks were obtained from (Patkar and Sharan 2018). In this paper, two separate yeast datasets were used for knockout signatures: one is the Kemmeren dataset, which is the one used here; the other is the smaller Reimand data (Reimand *et al.* 2010). To compare SIGNAL to the models of Patkar *et al.* we ran SIGNAL on the same datasets, and compared the results (Table 2). Since Patkar *et al.* proposed three different models, we always compared our predictions with their best model. Only the KPI set was used as signed data, as the other benchmark PPI set used by Patkar *et al.* (KEGG) was too small. For fair comparison, we followed Patkar’s cross-validation procedure by randomly splitting PPIs into 5 different groups, rather than the enzyme-based stratification reported above. SIGNAL shows improved prediction AUCs for the KPI set with an AUC of 0.98 (versus 0.77) for the Kemmeren dataset and an AUC of 0.97 (versus 0.83) for the Reimand dataset.

**Table 2.** SIGNAL performance comparison with previous state of the art sign prediction model.

Signed Interactions Dataset	Knockout signatures dataset	Model	AUC
*KPI*	Kemmeren	Patkar 2018, AllSP	0.77
*KPI*	Kemmeren	SIGNAL	0.98
*KPI*	Reimand	Patkar 2018, ASP	0.83
*KPI*	Reimand	SIGNAL	0.97

### 3.2 Knockout signature reconstruction

**Figure 3.**
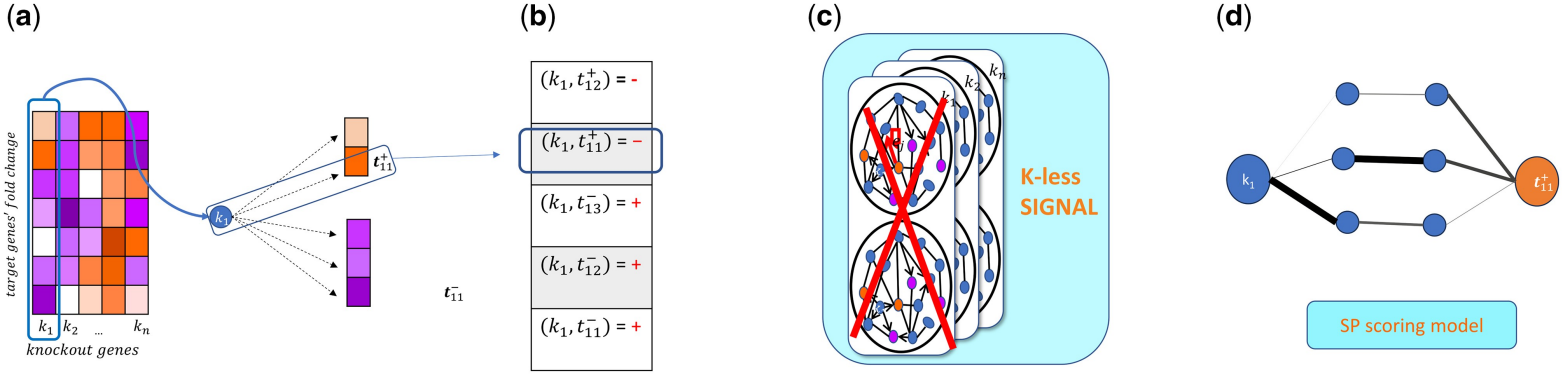
Overview of knockout signature reconstruction pipeline for a single knockout. (a) Knock out signature heatmap. Gene $k_{1}$ and its signature is taken as a binary vector (b) where upregulated genes are given a 0 and downregulated genes are given a 1. (c) All genes from the resulting vector are removed from SIGNAL features before running the algorithm, to prevent overfitting. (d) Shortest paths between gene *k* and target *t* are extracted, and their edge weight is assigned through SIGNAL. The *negativity score* is calculated as in Section 2.5.

We evaluated SIGNAL for its ability to reconstruct knockout signatures in *S. cerevisiae*, using the signed PPI network and the reconstruction model described in Section 2.5. The validation builds on the hypothesis that knockout signatures can be reconstructed from the signed interactome, since for any single knockout *k*, the targets which are significantly up (down) regulated are linked by a cascade of interactions to the source knockout, and the product of the signs in this path is the *inverse* (since the observed effect is caused by a knockout) of the final target response. To test this hypothesis, knockout-target pairs (*k, t*), were binarized into up-regulated and down-regulated sets (Fig. 3a and b), and SIGNAL was applied to predict edge signs in the interactome. For each pair, shortest paths were retrieved from the signed network and scored according to the fraction of negative paths (NegScore). To prevent overfitting, SIGNAL was trained with k-less propagations, in which all features involving k were excluded during training (Fig. 3c).

**Figure 4.**
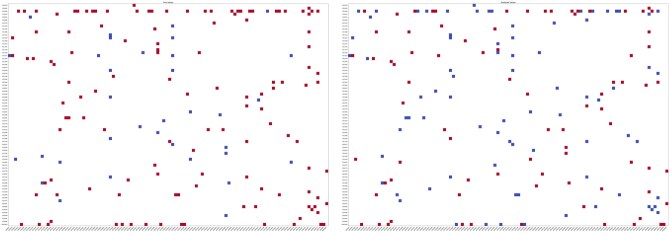
Knockout signature reconstruction of knockout-target pairs with at least 100 shortest paths connecting them inside the PPI network. *x* axis: knock out genes; *y* axis: target genes (genes whose expression has been altered by the knock out). Red represents a positive sign (upregulation), and blue a negative sign (downregulation). (a) Original signs of the knockout signature matrix; (b) Reconstructed sign, calculated with SIGNAL phenotype prediction model.

Steps (a–d) in Fig. 3 were repeated for all knockouts, and performance was evaluated by comparing reconstructed. *NegScore* vectors with the observed knockout signatures Across all knockouts, SIGNAL achieved area under the resulting precision-recall curve gave an AUC of 0.87. Figure 4 displays a heatmap of the *NegScores* vectors and compares it with the original (*k, t*) matrix heatmap. The figure shows that SIGNAL was able to reconstruct the sign of the knockout signature and to capture causal knock out-target relationships through the signal ratio of shortest paths, by correctly predicting the sign of differential expression of knockout targets.

### 3.3 Telomere length maintenance (TLM) phenotype reconstruction

The yeast telomere length maintenance (TLM, see Section 3, available as supplementary data at *Bioinformatics* online) dataset was used as a benchmark for evaluating the phenotype reconstruction capacity of SIGNAL (Section 2.5). Telomere length in yeast is tightly regulated by a dynamic equilibrium involving elongation and shortening mechanisms (Shachar *et al.* 2008, Ungar *et al.* 2009), guided by a small set of telomere binding proteins, together with a broader circuit of ∼500 TLM genes that interact with them (Peretz *et al.* 2022).

**Figure 5.**
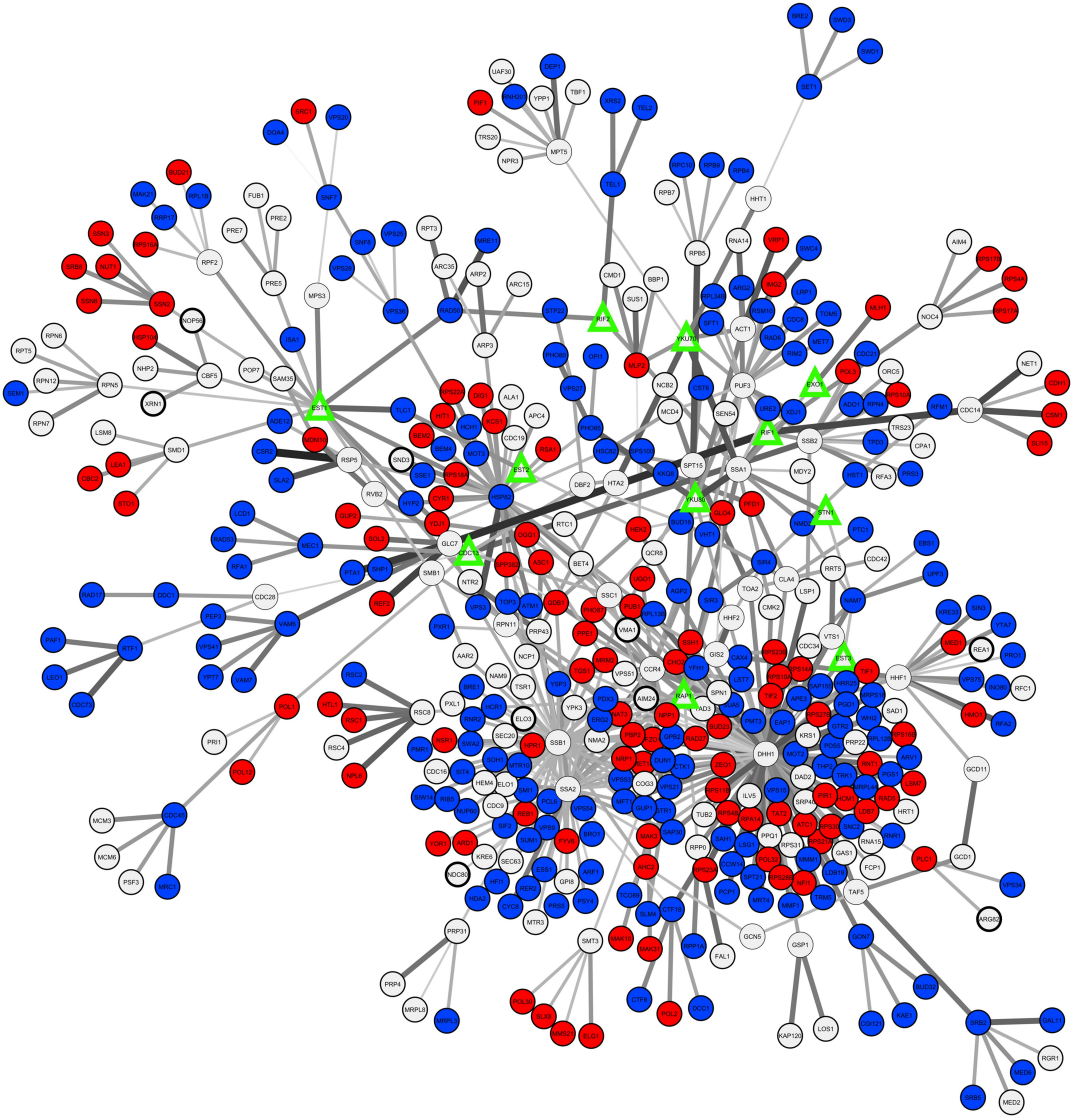
ANAT (Signorini *et al.* 2021) + SIGNAL TLM network: green triangles represent ANAT anchors, blue circles represent ANAT terminals with telomere shortening phenotype, red circles represent ANAT terminals with telomere lengthening phenotype; edge width is proportional to SIGNAL score. The anchored network was generated through the ANAT package on the Cytoscape software, and edge weights were uploaded on the same software to generate the representation.

The SIGNAL phenotype reconstruction model was applied to reconstruct shortening or lengthening phenotypes for 101 TLM genes and 11 telomere machinery genes (Fig. 5, Tables 1 and 2, available as supplementary data at *Bioinformatics* online). NegScores were computed for each pair of telomere machinery gene (anchor) and TLM gene (terminal) pair. The analysis focused on TLM mutants with a very strong phenotype (13 mutants with short phenotype, 5 mutants with long telomeres), and on 83 essential genes (50 short, 33 long phenotype), by building the phenotype reconstruction model connecting the genes with 12 telomere machinery genes, yielding a final dataset of 493 predictions (37 for short phenotype and 456 for long).

Evaluation by precision-recall analysis gave an area under the curve (AUPRC) of 0.8, indicating that SIGNAL accurately recapitulates TLM knockout phenotypes.

To visualize these results, a functional subnetwork anchored on telomere machinery genes and terminating on TLM genes was generated using the ANAT package in Cytoscape (Signorini *et al.* 2021) (Fig. 5). ANAT builds functional subnetworks linking source proteins to target proteins, providing a useful insight on the possible interactions of such a complex machinery. Edge weights from SIGNAL were overlaid, producing an interpretable map that highlights the network structure underlying telomere maintenance phenotypes.

### 3.4 Functional enrichment analysis

After predicting a SIGNAL score for every edge in our genome-wide PPI interaction network, we explored whether interactions with high SIGNAL scores (i.e. likely to be negative) tended to be enriched within a specific function. We calculated functional enrichment of 3412 predicted negative nodes across 1520 GO terms, a revealing significant (adjusted *P* value ≤.001), over representation in three main functional categories: (i) ubiquitination and protein degradation processes, (ii) RNA splicing machinery, and (iii) protein transport across intracellular membranes (Table 3). These results highlight that negatively signed interactions are not randomly distributed across the interactome. Interestingly, we observed significant enrichment of negative edges around key protein import receptors for both peroxisomes (Pex5/Pex7) and mitochondria (Tom20/Tom22), suggesting that negative interactions may play a crucial role in regulating organellar protein import. For a detailed analysis of these findings and their biological implications, see Section 4.1.

**Table 3.** Top enriched GO terms for negative interactions.^a^

Name	GO_ID	**-log10(A** **djPval)**
proteasome regulatory particle assembly	GO:0070682	11.5
U4/U6 x U5 tri-snRNP complex	GO:0046540	8.4
RSC-type complex	GO:0016586	8.2
poly(A) RNA polymerase activity	GO:1990817	5.4
spliceosomal complex	GO:0005681	4.9
**protein import into mitochondrial matrix**	**GO**:**0030150**	**4.9**
proteasomal ubiquitin-independent protein catabolic process	GO:0010499	4.4
Golgi to plasma membrane transport	GO:0006893	4.3
**protein import into peroxisome matrix, docking**	**GO**:**0016560**	**4.2**
proteasome core complex, alpha-subunit complex	GO:0019773	4.2
amino acid catabolic process to alcohol via Ehrlich pathway	GO:0000947	4.2
protein deubiquitination	GO:0016579	3.9
proteasome complex	GO:0000502	3.9
GINS complex	GO:0000811	3.9
5-phosphoribose 1-diphosphate biosynthetic process	GO:0006015	3.7
protein transmembrane transporter activity	GO:0008320	3.5
proteasome core complex	GO:0005839	3.4
proteasome regulatory particle, lid subcomplex	GO:0008541	3.4
U2-type prespliceosome	GO:0071004	3.4
protein refolding	GO:0042026	3.4
mismatched DNA binding	GO:0030983	3.3
COPI vesicle coat	GO:0030126	3.2
transcription factor TFIIIC complex	GO:0000127	3.2
generation of catalytic spliceosome for second step of splicing	GO:0000350	3.2
U1 snRNP	GO:0005685	3.1

a The table shows all terms found with an adjusted *P* value < .001. The functionally enriched terms can be broadly grouped in those related to (i) ubiquitination and protein degradation, (ii) splicing, and (iii) protein transport across intracellular membranes. Bold terms are those further described in the text.

**Figure 6.**
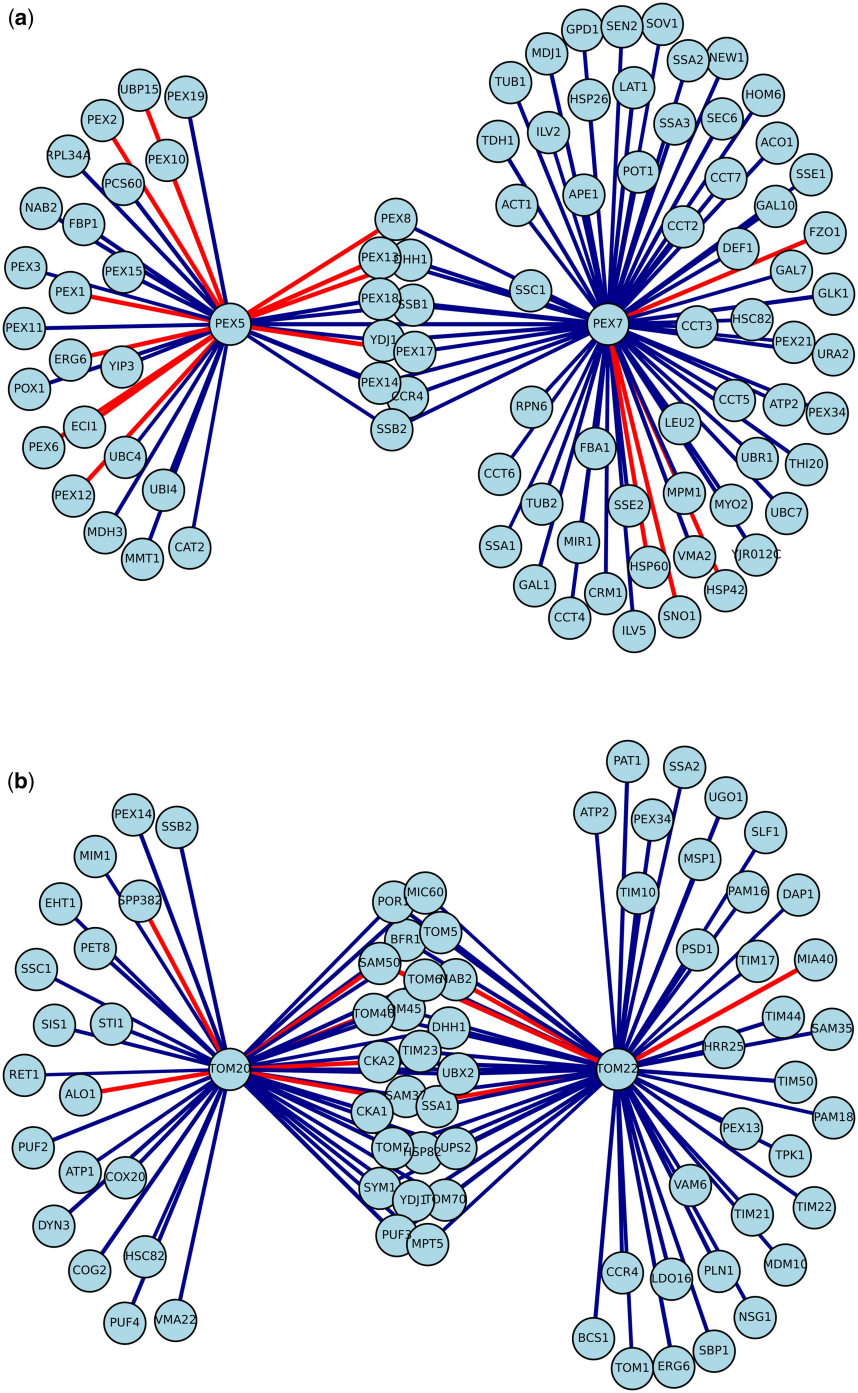
SIGNAL networks. (a) Peroxisome receptor SIGNAL network. Subnetwork of 101 PPI interactions between the neighbors of PEX5, and PEX7 peroxisome membrane receptors. (b) Mitochondrion outer membrane receptors SIGNAL network. Subnetwork of 101 PPI interactions between the neighbors of TOM20 and TOM22 mitochondrion outer membrane receptors. Red edges represent negative signal interactions. Interaction width represents SIGNAL intensity.

Peroxisomes are fundamental for life and highly conserved organelles across eukaryotes (Farre *et al.* 2019). They are crucial for cell metabolism, particularly in catabolic processes. Among the many metabolic processes that take place in the peroxisome are the oxidative degradation of purines, amino acids and long-chain fatty acids (Sibirny 2016). In particular, our results show an enrichment in interactions related to the import of proteins into the peroxisome matrix (GO:0016560). Such process is regulated by the interaction between peroxisomal membrane receptors, and PTS (Peroxisomal Targeting Signal) domains found on peroxisomal matrix proteins. In particular, PTS1 and PTS2 domains (which bind to three receptors Pex5, and Pex7, respectively) are responsible for the directing of peroxisome matrix proteins into the peroxisome matrix (Rottensteiner *et al.* 2004, Yuan *et al.* 2016). Building a SIGNAL subnetwork comprising PPIs adjacent to the receptors PEX5 and PEX7, showed a statistically significant enrichment (*P* value < .0001) of negative edges (Fig. 6a).

Mitochondria are double-membraned organelles that play a fundamental role in cellular energy metabolism by generating ATP through oxidative phosphorylation. In addition to energy production, they are involved in key processes such as apoptosis, calcium homeostasis, and the regulation of cellular signaling pathways (Nunnari and Suomalainen 2012). Similarly to the peroxisome case, results show that the protein import of proteins into the mitochondrial matrix (GO:0030150) is enriched for negative edges. For mitochondria, such process is mediated by the TOM (Translocase Outer Membrane) complex via the recognition and translocation of nuclear-encoded precursor proteins across the mitochondrial outer membrane into the intermembrane space or toward the inner membrane and matrix (Neupert and Herrmann 2007).

Within this complex, Tom20 and Tom22 act as primary receptors, by recognizing mitochondrial targeting sequences (MTS) contained in mitochondrial matrix proteins (Van *et al.* 1999, Yano *et al.* 2004). A SIGNAL subnetwork of PPIs adjacent to the outer membrane receptors Tom20 and Tom22 also exhibited a significant enrichment of negative edges (*P*-value < .0003, Fig. 6b). The cytosolic domains of both Tom20 and Tom22 possess a chaperone-like activity, helping to maintain substrate preproteins in an unfolded state and prevent their aggregation at the mitochondrial surface. Pex receptors, instead operate via a shuttling mechanism, entering the peroxisome matrix with their cargo before being exported and recycled to the cytosol (Sibirny 2016).

## 4 Discussion

In this paper, we presented SIGNAL, an algorithm for sign annotation of physical PPIs. We built our model based on a large collection of PPIs, using knockout signature data and known signed data from several databases and literature, and showed that it outperformed the current state of the art.

Our model is based on the simple premise that negative edges are less frequent in physical interactions, a straightforward yet impactful observation guiding our entire model. This was corroborated by the distribution of edge data found in the literature (Table 1), where the majority are in fact, positive.

Looking at the functional enrichment around protein import receptors of peroxisomes (Pex5/Pex7) and mitochondria, since a single negative interaction can reverse a pathway’s signal sign, one can hypothesize that the prevalence of negative interactions around these key import receptors may have evolved as a consequence of the need for fine-tuning the import process or its downstream effects. This mechanism could function like “adjusting water temperature in the shower,” where multiple, potentially finer, reversions of the signal allow for increasingly precise regulation until the desired final output (i.e. successful import, correct protein levels, regulated pathway activity within the organelle) is obtained.

While these findings highlight specific network properties associated with key protein import receptors, further experimental validation will be required to elucidate the precise mechanisms by which these negative interactions contribute to the regulation and fine-tuning of mitochondrial and peroxisomal protein import.

Even more interestingly, our model was able to predict the empirically observed phenotype from the TLM dataset in yeast with an AUC of 0.80. Interestingly, the experimental data labeled as the strongest phenotype was the one that yielded the best results, which has the double implication that both SIGNAL is able to accurately grasp and model underlying biological functions through the complexity of network interactions, and that stronger phenotypes are more easily classifiable and dealt with quantitatively. These evaluations aggregate the signs of multiple predicted edges along shortest paths, and therefore assess multi-edge sign prediction, not only edge-level accuracy.

SIGNAL harvests the power of network propagation algorithms, which have emerged as powerful tools for analyzing and extracting meaningful insights from network-based data (Cowen *et al.* 2017). These algorithms, which iteratively spread information through the network, are particularly adept at identifying patterns and relationships that might otherwise be obscured by the sheer volume and complexity of the data. Because propagation operates as a symmetric diffusion process, the influence that a perturbation exerts through an edge is independent of its orientation; consequently, SIGNAL can infer edge *signs* on undirected networks by modeling the relative propagation patterns of activating versus inhibitory interactions, without requiring prior knowledge of directionality.

### 4.1 Limitations

Naturally, scarcity of directionality on our interactions likely reduces the predictive power of our model, therefore future developments could address these limitations by coupling SIGNAL with orientation algorithms (Silverbush and Sharan 2019) to reconstruct signed and directed interactomes, and updating the model with new perturbation datasets for improved interactome coverage and new edge sign data for more refined integration of biological context.

Moreover, the assumption that all phosphorylations are activating, and all dephosphorylations are inactivating, although reasonable and used in previous works on the same task [e.g. the KPI in Patkar and Sharan (2018)], is an approximation. A similar caveat applies for ubiquitination of proteins, used for the UbiNet data. Finally, as more empirical evidence on interaction signs is gathered, this can be added to the model and is expected to improve its predictive power.

Biological interactions can be context-dependent, and the same protein pair may have different effects depending on cell type, condition, or post-translational state. SIGNAL provides a single, context-agnostic sign per interaction, reflecting a consensus effect supported by the perturbation data used during training. This static sign annotation is most useful as a general prior for large interactomes and for tasks such as propagation, pathway reconstruction, or network-based prioritization, where context-specific labels are often unavailable. In settings where regulation is strongly condition-dependent, context-specific models or multi-task extensions would be more appropriate (Purvis and Lahav 2013). Finally, SIGNAL focuses on sign, not causal direction, and can be combined with orientation or causal-inference methods when directional information is required.

## 5 Conclusion

In conclusion, we propose the SIGNAL model for sign annotation of physical PPIs. SIGNAL achieves accurate predictions by exploiting the imbalance of interaction signs in physical networks. It uses network propagation to identify negative interactions by their greater influence on gene expression changes compared to positive ones. With its superior performance, SIGNAL has the potential to impact the field of PPI network analysis and pave the way for new insights into cellular signaling pathways. As more data becomes available, SIGNAL’s predictive power is expected to increase further, making it an even more valuable tool for researchers studying cellular biology and disease mechanisms.

## Supplementary Material

btaf674_Supplementary_Data
