# Supplementary material for: Signing protein–protein interaction networks
Source: Bioinformatics. 2025 Dec 22;42(1):btaf674. doi: 10.1093/bioinformatics/btaf674 (PMC12781092; doi:10.1093/bioinformatics/btaf674)
Supplement: btaf674_Supplementary_Data [file btaf674_supplementary_data.docx]

**Signing Protein-Protein Interaction Networks**

**Supplementary information**

Lorenzo Federico Signorini, Martin Kupiec and Roded Sharan

1. Network Propagation

The network propagation algorithm was implemented in Python 3.9, using the *SciPy* and *NumPy* packages.

The implementation follows a linear iterative framework of the random walk with restart (RWR), and is available at <https://github.com/L-F-S/PPI_Network_Signer/score_edges.py>.

**Normalization of the Adjacency Matrix.** The PPI network is represented as an adjacency matrix $A$, where each entry $A_{ij}$ denotes the weight of the edge from node $j$ (source) to node $i$ (target). Prior to propagation, $A$ is column-normalized to form a transition probability matrix $W$, whose columns sum up to 1. Formally, a diagonal matrix $D$ is defined where $D_{jj}=\sum_{i} A_{ij}$​, is the sum of the *outgoing* edge weights from node $j$ of matrix, $A$ i.e, the total out-degree of node $j$. The normalized transition matrix is then obtained as:

$$\begin{aligned} W=AD^{-1} \end{aligned}(S1)$$

where $D$ is a diagonal matrix with $D_{ij}=0$ for $i\neq j$​, and $D_{jj}=\sum_{i} A_{ij}$ the sum of the *outgoing* edge weights from node $j$, i.e, the total out-degree of node $j$.

Adjacency matrix normalization is implemented using Compressed Sparse Row (CSR) matrix objects from the *scipy.sparse* library to represent $W, A,$ and $D^{-1}$ matrices. This representation enables efficient operations on very large and sparse graphs, such as the PPI network, as memory is only allocated for nonzero entries. Moreover, this formulation is robust for both directed and undirected graphs, since division by zero for isolated nodes is avoided by substituting zero column sums with 1.

**Propagation.** Given the normalized adjacency matrix $W$, a vector $\boldsymbol{P}_{0}$ is initialized with a set of seed nodes of interest (*e.g.*, a set of knockout genes from an experiment) with a value of 1, and all other nodes set to 0. $P_{0}$ represents the initial condition of all genes. This vector is then iteratively updated using the recurrence of Equation ‎3.2, reproposed here for readability:

$$\begin{aligned} \boldsymbol{P}_{\boldsymbol{i}}=\alpha W\cdot\boldsymbol{P}_{i-1}\boldsymbol{+}\left( 1-\alpha\right)\boldsymbol{P}_{0}\boldsymbol{\#}(S2) \end{aligned}$$

where $\alpha\in(0,1)$ is the propagation (or restart) coefficient, controlling the trade-off between retaining the initial signal and diffusing through the network, $\boldsymbol{P}_{0}$ is the starting point of the propagation, $\boldsymbol{P}_{i}$ is the propagated signal at timestep $\boldsymbol{i}$**.**

The iteration proceeds until convergence is achieved, which is defined as the change in propagated signal falling below a user-defined threshold ε, e.g.:

$$\begin{aligned} \left| \left| \boldsymbol{P}_{i}-\boldsymbol{P}_{i-1} \right| \right|_{2}<\varepsilon_{p}\#(S3) \end{aligned}$$

or until a maximum number of iterations is reached. The final output of the network propagation algorithm is a vector $P$ of propagated scores for all nodes in the PPI network.

Default parameters for network propagation used in this work were: $\varepsilon_{p}={10}^{-5}; \alpha=0.8; propagation iterations=100$

1. Knockout signatures data preprocessing

Knockout and perturbation signatures provide the causal data that underlie the SIGNAL model, linking a perturbed gene to the set of transcriptional changes it induces.

For *S. cerevisiae*, we relied on the dataset of (Kemmeren, et al., 2014), which measured transcriptional responses to 700 single-gene deletions using microarray profiling (see Introduction section ‎2.2.1.2). The dataset records 3017 differentially expressed transcripts, each annotated with the direction of change (up- or down-regulation).
For *H. sapiens*, genome-wide Perturb-seq profiles generated in K562 myelogenous leukemia cells (Replogle, et al., 2022) were used (see Introduction section ‎2.2.2). In this study, perturbations were screened by CRISPR interference (CRISPRi) and differentially expressed genes were identified using the Anderson-Darling test, corrected for FDR. Following the criteria in the original study, only a set of 1973 “strong perturbations” (and 2319 affected genes) were retained, ensuring that weak or noisy perturbations were excluded from downstream analyses.

Both yeast and human datasets were processed through a unified preprocessing pipeline, ensuring consistency despite their different experimental origins. Raw signature data were processed using the SIGNAL preprocessing routines in preprocessing_scripts/preprocess_pert_map.py:

1. **Gene ID mapping**: all gene identifiers were converted to Entrez Gene IDs to ensure consistency with the interactome. Unmappable IDs, duplicated IDs and IDs not present in the base network were dropped. In the yeast dataset, 135 unmappable target IDs and 53 target IDs not present in the base network were dropped. For the human dataset, 27 unmappable target IDs and 9 perturbation IDs were dropped, along with 129 targets not present in the base network.
2. **Thresholding**: the signature matrix was split into up and down target sets per perturbation using a species-specific absolute threshold on the expression change value. For yeast, a threshold of $\log_{2} FC=\pm1.7$ was used to define up vs. down regulated targets, following the original source. P-values had already been filtered at $p<0.05$by the original study. For humans, strong perturbations had been already been filtered at source, therefore the threshold for expression filtering was set to 0. Negative values were taken as down regulated and positive values as up regulated targets.
3. **Consistency filtering**: only perturbations with at least one up regulated target or one down regulated target were retained.

Applying these criteria yielded 235 knockout signatures and 1000 targets for yeast, and 1952 perturbations with 2168 targets for humans. After preprocessing, the signatures were serialized as two Python dictionaries, mapping each perturbed gene (KO/perturbation) to a set of up regulated or down regulated genes, respectively. This compact representation serves as the input format for SIGNAL, where each perturbation provides a causal-effect vector to be propagated through the PPI network. Dictionaries were saved as *pickle* binary files for downstream analysis.

1. The Telomere Length Maintenance (TLM) machinery in *Saccharomyces cerevisiae*

Telomeres are nucleoprotein structures that protect chromosome ends and are essential for genome stability. In yeast, telomere homeostasis depends on telomerase activity, DNA replication, and a wide range of cellular processes. Loss of proper telomere regulation results in progressive shortening or lengthening of telomeres, with downstream consequences for cell viability and genomic integrity.

Telomere length in yeast is tightly regulated by a dynamic equilibrium involving elongation and shortening mechanisms (Shachar, et al., 2008; Ungar, et al., 2009), guided by a small set of telomere binding proteins, together with a broader circuit of ~500 genes that interact with them (Peretz, et al., 2022) forming the telomere length maintenance (TLM) circuitry.

A first landmark large-scale screen in *Saccharomyces cerevisiae* identified genes whose deletion or perturbation leads to altered telomere length phenotypes (Askree, et al., 2004). These TLM genes encompass diverse cellular functions, including DNA replication and repair, chromatin remodeling, protein degradation, and RNA metabolism. Phenotypes were categorized into short or long telomeres, reflecting repression or enhancement of telomerase-mediated elongation.

This dataset was later extended and analyzed in several follow-up studies. (Shachar, et al., 2008) employed a systems-level approach to reconstruct the TLM gene circuitry, revealing functional modules within the telomere maintenance program. (Ungar, et al., 2009) performed a genome-wide screen for essential genes affecting telomere length, applying DAmP alleles to expand the TLM catalogue to essential processes. More recently, (Peretz, et al., 2022) provided a comparative analysis of telomere length maintenance circuits in fission and budding yeast, highlighting the evolutionary conservation and divergence of telomere regulation pathways. Together, these studies consolidated a catalogue of approximately 500 TLM genes, plus 12 telomere binding proteins (Table S1), which control, through a variety of mechanisms, telomere length in yeast, and which represent a reference resource for telomere biology and genome stability.

In this work, the TLM dataset is used as an independent benchmark to evaluate the predictive power of SIGNAL for reconstructing phenotype-associated subnetworks. Phenotypes are classified as short (loss of telomerase activity or impaired elongation) or long (enhanced elongation or de-repression of telomerase). In the original datasets, phenotype strength was manually annotated into very strong, strong, and slight categories. For the purposes of this work, analysis was restricted to TLM mutants with *very strong* and *DAmP* (essential genes) phenotypes only (Table S2).

All gene names were mapped to Entrez geneIDs, and unmappable genes were dropped from analysis. TLM gene overlapping telomere binding genes were removed, and the resulting collection was used to reconstruct a TLM subnetwork with ANAT 3.0, using TLM genes as terminals and telomere binding genes as anchors, and subsequently to evaluate the SIGNAL phenotype reconstruction model (defined in methods Section 2.5), where network-derived negativity scores were tested for their ability to predict TLM phenotypes.

**Table S1**. Telomere machinery genes.

| *Telomere Machinery Genes* |
| --- |
| EST1 |
| EST2 |
| EST3 |
| YKU70 |
| YKU80 |
| STN1 |
| CDC13 |
| EXO1 |
| RAP1 |
| RIF1 |
| RIF2 |

**Table S2.** Telomere Length Maintenance (TLM) genes and their associated phenotypes. VL = very long phenotype; VS = very short phenotype; DAmP Long = essential genes, long phenotype ; DAmP Short = essential genes, short phenotype.

| *Gene* | *Telomere Phenotype* |
| --- | --- |
| AAR2 | DAmP Long |
| ALA1 | DAmP Short |
| APC4 | DAmP Short |
| ARC15 | DAmP Short |
| ARC35 | DAmP Short |
| ARD1 | VL |
| ARP2 | DAmP Short |
| ARP3 | DAmP Short |
| BBP1 | DAmP Long |
| CAX4 | VS |
| CBF5 | DAmP Long |
| CDC16 | DAmP Long |
| CDC19 | DAmP Long |
| CDC34 | DAmP Short |
| CMD1 | DAmP Long |
| COG3 | DAmP Short |
| DAD2 | DAmP Short |
| FAL1 | DAmP Short |
| FCP1 | DAmP Short |
| GCD1 | DAmP Long |
| GPI8 | DAmP Long |
| HEM4 | DAmP Long |
| HRT1 | DAmP Short |
| ILV5 | DAmP Short |
| KRS1 | DAmP Short |
| LSM8 | DAmP Long |
| MCD4 | DAmP Long |
| MCM3 | DAmP Long |
| MCM6 | DAmP Long |
| MDN1 | DAmP Short |
| MRE11 | VS |
| MTR3 | DAmP Short |
| NAM7 | VS |
| NAM9 | DAmP Short |
| NCP1 | DAmP Long |
| NET1 | DAmP Short |
| NHP2 | DAmP Long |
| NOC4 | DAmP Short |
| NTR2 | DAmP Short |
| ORC5 | DAmP Short |
| PIF1 | VL |
| POP7 | DAmP Short |
| PRE2 | DAmP Short |
| PRE5 | DAmP Short |
| PRI1 | DAmP Long |
| PRP22 | DAmP Short |
| PRP31 | DAmP Short |
| SMD1 | DAmP Short |
| PRP4 | DAmP Short |
| PRP43 | DAmP Short |
| PRS3 | VS |
| PSF3 | DAmP Short |
| RAD50 | VS |
| RGR1 | DAmP Short |
| RNA14 | DAmP Short |
| RNA15 | DAmP Short |
| RNH201 | VS |
| RNR1 | VS |
| RPB5 | DAmP Long |
| RPB7 | DAmP Short |
| RPN12 | DAmP Short |
| RPN5 | DAmP Short |
| RPN6 | DAmP Short |
| RPN7 | DAmP Short |
| RPP1A | VS |
| RPS17A | VL |
| RPS31 | DAmP Long |
| RPT3 | DAmP Short |
| RPT5 | DAmP Short |
| RSA1 | VL |
| RSC4 | DAmP Long |
| RSC8 | DAmP Long |
| SAD1 | DAmP Long |
| SAH1 | DAmP Short |
| SAM35 | DAmP Short |
| SEC20 | DAmP Long |
| SEC63 | DAmP Long |
| SEN54 | DAmP Short |
| SMT3 | DAmP Long |
| SPN1 | DAmP Long |
| SRB8 | VL |
| TBF1 | DAmP Short |
| TEL1 | VS |
| TID3 | DAmP Short |
| TRS20 | DAmP Long |
| TRS23 | DAmP Short |
| TSR1 | DAmP Short |
| TUB2 | DAmP Short |
| XRS2 | VS |
| CDC9 | DAmP Long |
| CDC9 | DAmP Long |
| NCB2 | DAmP Short |
| HYP2 | VS |
| BET4 | DAmP Long |
| SIK1 | DAmP Long |
| CDC42 | DAmP Long |
| TAD3 | DAmP Long |
| RPP0 | DAmP Long |
| RFC1 | DAmP Long |
| YPP1 | DAmP Short |
| SWC4 | VS |
| RRP17 | VS |

# **References**

Askree, S. et al., 2004. A genome-wide screen for Saccharomyces cerevisiae deletion mutants that affect telomere length. *Proceedings of the National Academy of Sciences,* Volume 101, pp. 8658--8663.

Kemmeren, P. et al., 2014. Large-scale genetic perturbations reveal regulatory networks and an abundance of gene-specific repressors. *Cell,* Volume 157, pp. 740--752.

Peretz, I., Kupiec, M. & Sharan, R., 2022. A comparative analysis of telomere length maintenance circuits in fission and budding yeast. *Frontiers in Genetics,* Volume 13, p. 1033113.

Replogle, J. et al., 2022. Mapping information-rich genotype-phenotype landscapes with genome-scale Perturb-seq. *Cell,* Volume 185, pp. 2559--2575.

Shachar, R. et al., 2008. A systems-level approach to mapping the telomere length maintenance gene circuitry. *Molecular systems biology,* Volume 4, p. 172.

Ungar, L. et al., 2009. A genome-wide screen for essential yeast genes that affect telomere length maintenance. *Nucleic acids research,* Volume 37, pp. 3840--3849.
